# Closing the Feedback Loop: Improving Communication of Findings With Aboriginal Research Participants Through Participatory Action Research

**DOI:** 10.1111/hex.70798

**Published:** 2026-08-14

**Authors:** Stephanie Smith, Tania Harris, Kelli Savietto, Melanie Robinson, Glenn Pearson, Stephen Paull, Yvonne C. Anderson

**Affiliations:** ^1^ Curtin Medical School, Faculty of Health Sciences Curtin University Bentley Western Australia Australia; ^2^ Child and Adolescent Community Health Child and Adolescent Health Service Perth Western Australia Australia; ^3^ The Kids Research Institute Australia, Perth Children's Hospital Nedlands Western Australia Australia; ^4^ Health Consumers' Council WA Mount Lawley Western Australia Australia; ^5^ Kelli Savietto, Indigenous Artist & Graphic Designer Boorloo (Perth) Western Australia Australia; ^6^ Child and Adolescent Health Service Perth Western Australia Australia; ^7^ Department of Paediatrics: Child and Youth Health, Faculty of Medical and Health Sciences University of Auckland Grafton Auckland New Zealand

**Keywords:** Aboriginal and Torres Strait Islander Peoples, consumer and community involvement, feedback, healthy lifestyle, participatory action research, qualitative

## Abstract

**Background:**

Communication of research findings often lacks appropriately tailored messaging to meet participants' expectations and needs. Guidelines for communicating with Aboriginal and Torres Strait Islander Peoples exist but are not widely applied in practice. An April 2024 workshop with 29 Aboriginal advisors explored barriers, enablers and mitigation strategies for locally adapting an evidence‐based Healthy Lifestyle Program. A book was recommended as a culturally appropriate mode to share workshop findings with participants. This study explored Aboriginal Elders' experiences of receiving study findings, guidance for researchers and produced a user‐friendly book reporting the cultural and place‐based program considerations.

**Methods:**

Participatory action research guided the co‐creation of the book. Nine Elders from the Healthy Lifestyle Program Cultural Advisory Group, who had participated in the April 2024 workshop, took part in the June 2025 workshop. An Aboriginal facilitator conducted the workshop, supported by three non‐Aboriginal researchers. Previous workshop findings were summarised. The workshop was audio‐recorded, transcribed verbatim and data were analysed using reflexive thematic analysis. The Cultural Advisory Group were involved throughout the research and book development.

**Findings:**

An overarching theme of *knowledge sharing* across research partnerships, communities and families connects the two themes identified. *Working in partnership* outlines the need for Aboriginal participants' contexts and lived realities to be recognised by researchers, and the group's previous and current research experiences. *Leaving a legacy* highlights the importance of the book as a lasting record, incorporating artwork and multiple formats to broaden accessibility and reach to diverse audiences, including younger generations, ensuring that community voices and values continue to guide research.

**Conclusion:**

Prioritising Aboriginal and community voice in communicating findings is critical to genuine partnership in research. Consumer and community involvement within participatory action research is important to effectively implement unique research recommendations. Through this work, a book was co‐created, hard copies were made available to the April 2024 participants, and an online version is available. Future research recommendations are provided. Practical tips are also provided to support researchers in building trust and collaborating effectively with Aboriginal participants/community members.

**Lived Experience or Public Contribution:**

An established Cultural Advisory Group (10 Aboriginal Elders) were involved throughout the research using a participatory action research approach, embedding consumer and community involvement. In their advisory role, the Elders guided key decisions, including endorsing the development of the book to report findings from the April 2024 workshop. The group reviewed the study findings and co‐created the book with the research team. Three Elders were nominated for additional oversight, with two reviewing the final manuscript and book, and one reviewing the book's Acknowledgement of Country. This work was also supported by a representative consumer organisation, Health Consumers' Council WA.

## 1 Introduction

International guidelines to conduct ethical research, such as the Declaration of Helsinki, recognise that all research participants should be given the opportunity to be informed about a study's results [1]. Participants contribute their time and expertise to improving knowledge and guiding service improvements for future groups. Reporting findings back to those who have contributed is not only basic courtesy but also an ethical imperative and good practice [2, 3]. The importance of reporting research to participants has been widely endorsed in research communities and should be considered routine practice [4, 5].

Within Australia, the National Health and Medical Research Council provides guidelines on feedback mechanisms to research participants. These include providing multiple formats ensuring participant preferences are taken into consideration when choosing a reporting method [6], which may include creative outputs [7]. Consumer and community involvement (CCI) is a term used within Australia to describe consumers and community as partners in health and medical research [8, 9]. ‘Consumer’ and ‘community’ are terms often used interchangeably [8]. Consumers are defined as individuals with lived experience or people who represent the views of a consumer organisation or community [8, 9, 10, 11]. There has been considerable progress in partnering in research [12] including methods such as research buddies and advisory groups [8] to ensure that research remains relevant to end‐users [13, 14] and guides dissemination practices [15]. There is currently a renewed call for action to implement meaningful changes in CCI practice to effectively establish genuine Aboriginal and Torres Strait Islander partnerships [8].

It has long been known that most participants involved in research want to receive study findings upon a study's completion [5, 16, 17]. Similarly, researchers are also keen to share findings. However, reported barriers include limited time and resources for communication and dissemination, as well as insufficient knowledge and skills related to sharing findings [17, 18]. These barriers are further exacerbated in research with Aboriginal and Torres Strait Islander Peoples as it may require extended timeframes to establish partnerships between researchers and the community, which often conflict with standard funding timelines [19, 20]. Sharing findings with participants helps build trust and shows respect, demonstrating that participants' voices are heard and valued [21, 22]. However, findings are not always delivered in a way that participants prefer or understand. Previous research has provided insights from participants on the appropriate mode, timing, context, how to share sensitive findings and the role of community partners in dissemination [23, 24]. Tailoring research messaging to ensure relatability and to meet participants' varying expectations should therefore be considered [2, 17].

Aboriginal and Torres Strait Islander Peoples are Australia's First Peoples and have the world's longest continuous culture [25]. Country is a statement of identity, spirituality, belonging, law, culture, knowledge, responsibility and connection to the land, water and sky through ancestral ties and family origins [26, 27, 28, 29]. Language groups are cultural, linguistic and kinship collectives of Aboriginal people that share language or dialect and connections through one or more Countries [30, 31]. Colonisation impacted Aboriginal and Torres Strait Islander Peoples health and well‐being through displacement of Country and being moved to missions and reserves, which disrupted culture, language and knowledge systems and caused the separation of families and communities [28, 32]. Colonisation also embedded systemic racism and inequality into education, housing and justice systems which continue to shape health and well‐being today [24, 33, 34, 35]. It is crucial that Aboriginal and Torres Strait Islander Peoples voices shape and guide decision‐making about research feedback formats that are meaningful and relevant to them, and support improved equitable healthcare provision and outcomes [36, 37]. Western Australia (WA) is Australia's largest state, characterised by vast geographic diversity, including metropolitan, regional and remote communities. In 2021, 120,000 people in WA identified as Aboriginal and Torres Strait Islander, representing 4.4% of the population [38]. Due to inequalities rooted in the impacts of colonisation, many Aboriginal and Torres Strait Islander Peoples continue to experience worse health outcomes compared with background population data [39]. An area that is a focus in the WA Sustainable Health Review is childhood obesity [40].

### 1.1 The Healthy Lifestyle Program

The Healthy Lifestyle Program was designed for children and young people aged 4–16 years (above a healthy weight) and their families who want to achieve a healthy lifestyle change [41]. Aboriginal and Torres Strait Islander families and those from socioeconomically disadvantaged backgrounds were prioritised, given the over‐representation of these population groups in obesity statistics [42]. ‘Healthy Lifestyle Program’ and ‘program’ will be used interchangeably. The program was a pilot being conducted within the East Metropolitan Health Service (EMHS) catchment area in Perth by Child and Adolescent Community Health in collaboration with Curtin University and The Kids Research Institute Australia. It included a multidisciplinary team with clinical and research streams, with CCI working in partnership throughout the research. The CCI groups include Aboriginal Elders (individuals who hold cultural authority within their family and traditional lands—each community has a number of Elders representing different groups of that community) [43] and families with lived experience of children living above a healthy weight and lived experience of the health systems. The program was informed by Whānau Pakari (which means healthy, self‐assured whānau [wider family unit]) [44]. Whānau Pakari is a community‐based New Zealand (NZ) healthy lifestyle program with a 15‐year evidence base, co‐created with community and evaluated based on international recommendations for care of children and young people affected by obesity [44, 45, 46, 47].

The pilot program was funded for 22 months and undertook assessments at baseline (program entry), follow‐up (5–10 months from baseline) and completion (11–15 months from baseline), screening for weight‐related comorbidities. Weekly activity and education sessions for participants were delivered over a 6‐month period. Referrals were accepted from all health professionals within the identified communities, including child health nurses, school health nurses, Aboriginal and Torres Strait Islander health practitioners, Derbarl Yerrigan Health Service (Aboriginal Community‐Controlled Health Organisation) and other health professionals [41]. Self‐referrals were also accepted. The full program and research protocol are reported elsewhere [41].

### 1.2 Workshop Study

As part of the program's pre‐implementation phase, a workshop was conducted with Aboriginal Advisors (29 attendees, 28 being Elders) in April 2024, exploring barriers, enablers and mitigation strategies for adapting the program to the Perth context and applying cultural and place‐based considerations [41]. Further details of this qualitative study are reported elsewhere [48]. During this workshop, participants recommended that the findings be reported and delivered back to them in the format of a book.

In the process of this, uh, meeting and everything that's written down…after, would it be able to turn into a book so that we can have a record of today? I would like to have a book of this, what's been spoken, what's been written…as a book that we can have in our household to go by and you know, and to share with families around us…There's so many families here and that can be shared with the family communities around Perth and that Country.

(Workshop Advisor, April 2024)

The ‘Cultural Advisory Group’ (10 Elders), also referred throughout as ‘the group’, ‘member(s)’ or ‘Elder(s)’ were recruited from the April 2024 workshop to work in partnership with the program as it evolved. In December 2024, the book recommendation was discussed and identified by the group as the preferred feedback approach for the 29 original workshop participants.

The current study aimed to (1) explore Aboriginal Elders' experiences of receiving study findings and provide researcher guidance; (2) develop a culturally appropriate, relatable book to close the feedback loop for the previous Aboriginal workshop participants.

## 2 Methods

### 2.1 Design

This study utilised participatory action research (PAR), which emphasises that those being researched should be partners in the research process, shaping the research topic, contributing to data collection and analysis and determining actions arising from the findings [49]. PAR is an effective method for engaging Indigenous people and communities in research, providing communities with control over the research processes and outcomes [50]. CCI was incorporated within PAR. The Cultural Advisory Group had participated in the previous and current workshop studies, and within their advisory role approved the book recommendation as a suitable feedback approach and further guided on the findings and the book development throughout the research process.

The qualitative component of this study included a 1.5‐hour workshop embedded into an existing Cultural Advisory Group meeting. Given the group's prior experience of participating in a workshop [48], this approach was chosen.

### 2.2 Participants

The Cultural Advisory Group was purposively sampled due to their advisory role and prior participation in the April 2024 workshop. The April 2024 workshop included 29 Aboriginal advisors, from which a smaller group of Elders formed the Cultural Advisory Group. Participation in the April 2024 workshop was a pre‐requisite for inclusion. The Cultural Advisory Group has 10 members that meet every quarter or as required to discuss the program's progress and research considerations, providing guidance on cultural appropriateness and the cultural safety of the program and participants.

### 2.3 Recruitment

T.H. and S.P. contacted the participants by phone, provided an overview of the Participant Information and Consent Form (PICF) and addressed any queries about participation. The PICFs were posted to all Elders prior to the workshop in May 2025. Ten members of the Cultural Advisory Group were invited to participate.

### 2.4 Data Collection

Participants were given time at the start of the workshop to settle in and reconnect. Refreshments and lunch were provided. Informed written consent was obtained from all participants at the start of the workshop, alongside demographic information. Participants also had the opportunity to ask any further questions about the research before the workshop started. TH facilitated the workshop, supported by S.S. and Y.C.A. as co‐facilitators, and S.P. presented the April 2024 workshop findings. The Marketing and Communications Coordinator from Health Consumers' Council WA (HCCWA) attended to provide guidance on developing the output. A semi‐structured workshop schedule was followed, with participants preferring to work as one group. Table 1 provides the workshop schedule of the questions and prompts. Permission was obtained to record the workshop for analysis purposes. A brief introduction to the study, along with sharing group guidelines for participation followed. The workshop included exploring the group's experiences with participating in research and receiving feedback, a presentation on the research findings from the April 2024 workshop study to review the potential book content [48] and reviewing how to report findings in the chosen format. An overview of the previous workshop themes was provided at the tables for reference after the presentation for the group to refer to as needed. Materials from other research studies, including a poster with a QR code linking to an oral presentation, another poster reporting study findings and a booklet, were shared with the group as format examples and prompts for discussion. The Cultural Advisory Group members were reimbursed for their time based on locally approved rates.

**Table 1:** Workshop schedule.

Background question	Can you tell me about your previous experiences of receiving feedback on study findings?
	Prompts: Were the study findings shared? Were the study findings understandable?
Presentation	*Presentation of the research findings from the April 2024 study*
Key questions	*Theme summary of the April 2024 workshop provided*
	*Butcher's paper and pens available to participants*
	*Examples of formats provided*
	Please discuss how the study findings should be presented.
	Prompts: Certain findings to be included? Artwork to include?
	What guidelines or recommendations do you suggest for researchers in reporting research findings back to Aboriginal participants?
	Prompt: Tips for researchers
Closing question	Is there anything else we have not yet discussed that you would like to add?
	Prompt: Is there anything else that is important to the development of the output or sharing findings with participants?
Probing questions	Can you tell me more about…?
	Can you give an example of…?
	Can you elaborate on that…?

*Note:* Italicised text indicates information and materials presented to participants during the workshop.

### 2.5 Data Analysis

The workshop was audio‐recorded and transcribed verbatim by a transcription service, with a confidentiality agreement in place. The transcript was reviewed against the recordings several times by S.S. for accuracy. Data were analysed using reflexive thematic analysis [51]. The method's flexibility was chosen to honour Aboriginal knowledges and to prioritise participants' perspectives [52, 53]. Data were managed in NVivo 14 [54]. S.S. followed a recursive process, moving back and forth between phases of analysis, with phases often blending [51, 55]. The analysis was positioned between semantic and latent interpretation. While remaining closely grounded in participants' accounts, interpretative engagement with the data was important for understanding the cultural context and broader significance of participants' experiences, relationships and perspectives. Consistent with Braun and Clarke's reflexive thematic analysis [55], this analytic positioning recognises that semantic and latent meanings exist on a continuum and that analytic depth was guided by the research question and purpose. All authors and the Cultural Advisory Group reviewed the themes. Table 2 provides an overview of the analytical process, description and reflexivity [55].

**Table 2:** Analytical process, description and reflexivity.

Reflexive thematic analysis phases	Description
Data familiarisation and writing familiarisation notes	S.S. led the analysis process and was immersed in the data.
	The transcript was checked against the audio recordings within the transcription software and against the original recordings.
	The transcript was read and re‐read several times.
	Annotations were made in NVivo 14 and included notes and questions on the meaning and significance of what was being said. Notes also included areas of potential interest and ideas to explore further in coding, and any responses to the data.
	The summary notes made with the group during the workshop were also referred to and discussed between S.S., T.H. and Y.C.A.
Systematic data coding	The entire dataset was coded systematically and inductively, encompassing three rounds of coding. A recursive approach, utilising open coding, was employed.
	Coding labels were used to capture the essence of statements.
	Codes were both semantic and latent.
	A list of codes was formed.
Generating initial themes from coded and collected data	Codes were sorted for initial and developing themes.
	Merging codes of similar topics started.
	The data were reviewed to identify potential themes within the code.
	Memo's within NVivo 14 were used to record why codes were grouped together or amended and further reflections on the relationships of themes.
Developing and reviewing themes	All references within each theme were reviewed to ensure consistency, and the validity of individual themes in relation to the dataset was assessed.
	Themes were checked in relation to coded extracts (Level 1) and the entire dataset (Level 2).
	Coding stripes in NVivo 14 were useful for visually reviewing pattern identification.
	Themes were further developed through either being let go, merged or renamed.
	All processes were captured in memos within NVivo 14.
	Developing themes were discussed between S.S and Y.C.A.
Refining, defining and naming themes	An informative name for each theme was selected.
	Short descriptions of each theme were written.
	S.S. reviewed the themes with T.H., S.P. and Y.C.A., who supported reflexive discussions.
	S.S. presented the initial themes to the Cultural Advisory Group for their insights at a follow‐up meeting in October 2025 which K.S. attended.
	Whilst feedback in reflexive thematic analysis is not a validation strategy, some of the group stated that the themes had captured what they had said appropriately.
	Two members were nominated to review the manuscript prior to journal submission to ensure cultural appropriateness.
Writing the report	The analysis process continued in the writing of the findings.
	Some initial main themes were dropped to subthemes and others merged.
	Vivid and compelling quotes were used to illustrate each theme.
	The two nominated Cultural Advisory Group members reviewed the manuscript prior to journal submission with S.S. and T.H.

Rigour was supported through ongoing researcher reflexivity, iterative engagement with the dataset, documentation of analytic decisions and collaborative discussions to enhance interpretation, coherence and the trustworthiness of the findings.

### 2.6 Positionality

The research team aspires to conduct research that is by the community, for the community [41]. It involves diverse researchers and advisors from transdisciplinary backgrounds, with a commitment to strengths‐based research that prioritises and privileges the voices of children, young people and families alongside the community [41]. The group have a commitment to working towards health equity and working in genuine meaningful partnership [41].

The team holds expertise and capability in Aboriginal health, child health, CCI, graphic design, health systems change and implementation, implementation science, mixed‐methods research, nursing, paediatrics and strengths‐based community healthy lifestyle program delivery.

S.S. is a Senior Research Fellow on the Healthy Lifestyle Program and an experienced qualitative researcher specialising in health experiences, evaluation of health service delivery, CCI, health psychology, implementation science, qualitative research and mixed‐methods. S.S oversaw the research, co‐facilitated the workshop, conducted the analysis and oversaw the book development, supported by the team. T.H. is a Bundjalung woman (Queensland) and is the Engagement Manager, Aboriginal Engagement Lead at HCCWA, working with consumers and facilitating consumer and community events and workshops. T.H. facilitated the workshop and supported the book development. K.S. is an Indigenous artist and graphic designer of Nyikina and Yawuru heritage from the Kimberley region of WA. K.S. attended follow‐up meetings, interpreted group feedback for the artwork and created the artwork and book. M.R. has connections to Ngarinyin and Gidja Country and is Director in Aboriginal Health at Child and Adolescent Health Service. G.P. is Director First Nations Strategy and Leadership at The Kids Research Institute Australia. M.R. and G.P. oversaw the research and are part of the Aboriginal Governance Team with T.H. S.P. is a PhD candidate at Curtin Medical School, Curtin University, and medical registrar in general paediatrics at Child and Adolescent Community Health. The April 2024 study formed part of S.P.'s PhD. S.P. presented the April 2024 study findings on program adaptation to the Perth context and contributed to the book development. Y.C.A. is a paediatrician and professor of paediatrics and child health with experience working with marginalised populations and multidisciplinary teams, and in implementing a successful, translatable Healthy Lifestyle Program. Y.C.A. co‐facilitated the workshop and oversaw the project. S.S., S.P. and Y.C.A. had undertaken multi‐phase cultural security training as part of the pre‐implementation of the Healthy Lifestyle Program.

## 3 Findings

Nine members of the Cultural Advisory Group participated in the workshop. Table 3 outlines the demographics of the Cultural Advisory Group based on the Australian Bureau of Statistics cultural and ethnic groups and gender characteristics [56, 57], noting the preferencing of Country, language group and Aboriginal on request of the Elders. Most participants were female, and all were over 60 years. Most identified with different parts of the Noongar Country (South‐West region of WA and includes the Swan River region) [58] and some with Yamatji Country (Mid‐West region of WA) [30] with a range of language groups.

**Table 3:** Cultural Advisory Group demographics.

Demographic characteristics	Number (%)
Gender	
Female	7 (78)
Male	2 (22)
Age	
60–69	3 (33)
70–79	6 (67)
Ethnicity	
Aboriginal Australian	6 (67)
Aboriginal Australian, Irish, Chinese	1 (11)
Aboriginal Australian, Italian	2 (22)
Language group/Country	
Ballardong/Noongar	1
Goreng/Noongar, Yamatji	1
Menang/Noongar	1
Wagyl Kaip/Noongar	1
Wardandi/Noongar	1
Wajarri/Yamatji	2
Whadjuk/Noongar, Yamatji	1
Wiilman/Noongar	1

**Figure 1:**
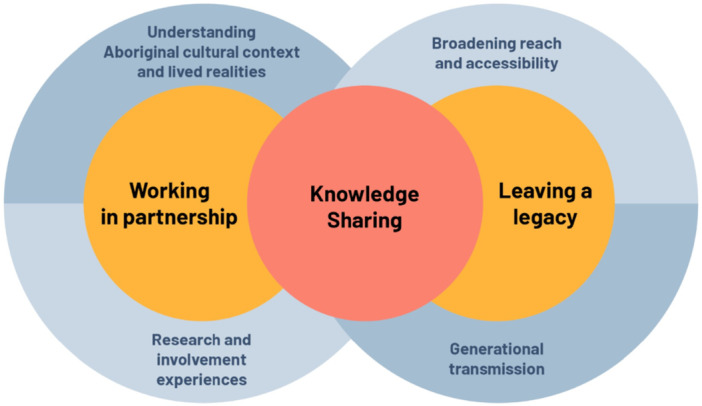
Overarching theme, themes and subthemes.

An overarching theme of *knowledge sharing* across the research partnership, communities and families connects the two themes and subthemes. Each theme reflects different but important dimensions of these relationships. *Working in partnership* recognises the need for researchers to understand Aboriginal participants' contexts and lived realities and draws on the group's previous and current research experiences. Partnership is positioned as a relational and reciprocal process through which knowledge is grounded in cultural accountability and shared within research interactions. *Leaving a legacy* highlights the importance of artwork and multiple modes of dissemination to engage diverse groups and generations, as well as innovative ways to disseminate the book within the community, ensuring that the next generation continues to guide research. This extends knowledge sharing beyond the immediate research context and into future generations across communities and families. Figure 1 shows the connections between the overarching theme, themes and related subthemes. The colours in Figure 1 link to the co‐created book, artwork and artwork story, developed through the research process: pink (*Knowledge Sharing*) represents the mind; orange represents Elders as holders of knowledge and story, and blue represents the river through which knowledge flows [59].

### 3.1 Working in Partnership

This theme positions research partnerships as contingent on cultural accountability. Cultural accountability refers to the responsibility of researchers to recognise cultural context, lived realities, connection to Country and to engage transparently and reciprocally with Aboriginal community members. Previous and current research experiences shape expectations of trust, transparency and communication, highlighting that partnership is a relational and ongoing process.

#### 3.1.1 Understanding Aboriginal Cultural Context and Lived Realities

Participants emphasised cultural understanding as essential to respectful and meaningful involvement in research, highlighting that without recognition of lived realities and cultural context, meaningful partnership is difficult to achieve. Accounts of healthy lifestyle were framed through the ongoing impacts of colonisation, from changes to traditional foods to access to housing, which continue to impact health and well‐being.

We just want yous to be aware exactly where we're, we're coming from in regards to our health, the food we grew up on, how healthy we were as young people to where living in this society now has turned that around and taken away our foods that we grew up on.

(Elder Female 1)

And the government said they looked after the Aboriginal people. All they gave them was a‐a golden syrup, sunshine milk, flour, sugar.

(Elder Female 6)

So that they [Aboriginal families] will be waiting and waiting and waiting [for housing]…And they get served last. Like we've got served last in the same process as following on behind me.

(Elder Female 3)

Recognition of Country was a fundamental aspect of participants' cultural identity and belonging and was identified as important to acknowledge in research practice. Two Elders had also provided their language groups and Country on the demographic forms next to ‘Aboriginal Australian’, and one stated ‘Aboriginal first’. The following was mentioned in a side conversation during the workshop.

[To research member] I'm just saying, that's where they should have [on the demographic form] their Aboriginal groups to identify what Country.

(Elder Female 5)

This illustrates how standardised demographic categories can inadvertently fail to capture the heterogeneity of Aboriginal identity. Participants actively shaped how they wished to be represented in research, emphasising language group and Country.

Understanding cultural context was important for preventing barriers to effective partnership and collaboration, ensuring all parties were aligned.

So even that understanding around us as a group…in regards to how we grew up, and we're sitting around the table with you now. But if you got no idea where we come from‐how‐how do you know what we've got to, um, what we're giving you is workable?

(Elder Female 1)

Cultural training was highlighted as crucial for researchers in understanding the cultural context and lived realities of Aboriginal people, emphasising the need for greater attention to linguistic diversity.

Well, you know when your fellas [researchers] do your cultural awareness training, do yous do anything around that, around the difference between Noongar [people are the traditional owners to the South‐West of WA] and Yamatji [people are the traditional owners to the Mid‐West region of WA] and?…And they talk about the language, language of different language. Yeah?

(Elder Female 1)

Collectively, these accounts construct cultural understanding as necessary for culturally safe and authentic partnership.

#### 3.1.2 Research and Involvement Experiences

Participants' accounts illustrate how experiences of research involvement influence expectations of trust, transparency and communication, positioning these elements as integral to how research partnerships are understood and evaluated. For most participants, the April 2024 workshop and this workshop study were their only experiences of participating in research. Two participants with prior research experience highlighted both the benefits and challenges of research collaboration. Positive experiences were associated with receiving ‘appropriate’ (Elder Female 1) information and feedback that aligned with participant preferences, reinforcing trust and supporting engagement.

Having sessions like this where we were shown on, um, on a screen like this throughout the process, and then at the end of the process, a booklet with a DVD in it.

(Elder Female 1)

In contrast, experiences of inaccurate reporting and poor communication undermined trust and impacted intended meaning.

So, you know, that's what I find sometimes. It's not giving the true picture of what's been said. So, whoever's doing the writing up, that will put it into changing the wording and that, you know, so it's not coming up as strong as what you're wanting it to be. And the whole message is lost.

(Elder Female 2)

Regarding previous involvement experiences, challenges with communication and documentation further shaped expectations, with participants highlighting how incomplete or unclear records limited their ability to meaningfully engage and retain information.

The meetings were huge, like really huge…I used to take my own minutes at the meetings…But I wasn't the minute taker…The minutes that we usually got before the next meeting didn't really cover everything that was discussed. Too big. The subject was too big…Then the group just fell apart.

(Elder Female 1)

Within the current partnership, requests for accessible formats, such as providing presentation materials, further highlight expectations for transparency and inclusion in research processes.

…can you put it into a PowerPoint presentation for us? [printout].

(Male Elder 1)

Ongoing feedback and transparent communication were noted as essential to sustaining partnership, with participants emphasising the need for reciprocal exchange rather than one‐sided input. Time, pacing and space for reflection were also identified as critical to meaningful engagement.

See you guys run everything, and you want our input into everything. But I don't feel like you're giving us the same…feedback, in regards to how yous are travelling…But it shows up sometimes when you start rushing around, and you're trying to do too much…that day when we had the big family [April 2024 workshop], when everyone came together, um when we was rushing from that table‐to‐table stuff, I would have liked more time on that. Because sometimes I don't take things [in]. There are days when I don't take a lot in, and that was one of those days.

(Elder Female 1)

The importance of direct and timely communication was also highlighted, reinforcing expectations of openness and accountability within the partnership. The following quote highlights the value of transparency in the partnership by using an idiom that emphasises addressing matters directly rather than avoiding or delaying the main point.

No beating around the bush (laughing).

(Elder Female 7)

Participants' involvement across multiple advisory groups and committees further shaped expectations of clarity and identity within research partnerships. The development of a distinct visual identity for the program was proposed to strengthen connection, ownership and recognition of their involvement.

Yeah. So, you know, like, with us being Elders, sometimes we think, ‘Oh, well.’ When you send us out messages, we think, ‘Now, which one is this?’

(Elder Female 5)

No, we want our own [t‐shirts/logo].

(Elder Female 1)

Particularly, for this group.

(Elder Female 2)

These accounts position trust as actively shaped by experiences of research involvement, where transparency, accurate representation and ongoing communication underpin meaningful and sustained research partnerships. They also reflect the importance of identity and visible markers of representation in how partnerships are recognised and valued.

### 3.2 Leaving a Legacy

This theme emphasises the importance of making the book widely available and accessible in multiple formats, promoting inclusivity and extending its reach across family groups and communities. The book was not just an output, but a resource intended to carry knowledge and community voice across generations. Central to this was the aspiration to inspire future generations to become involved in research, carrying forward the legacy of involvement.

#### 3.2.1 Broadening Reach and Accessibility

There was a shared understanding that the book must be accessible, adaptable and responsive to diverse community needs, with the hard‐copy book being the most favourable format for presenting the research findings.

We'll get a book.

(Elder Male 2)

Concerns regarding the potential size of the book further reflect participants' emphasis on usability and engagement. The three themes from the previous study [48] were presented along with several quotes. Some questioned whether the book might become too lengthy. This sentiment is captured humorously in the following quote, which compares the anticipated content to a well‐known expression for something grand in scale.

[The book] As big as Ben Hur by the sounds of it.

(Female Elder 4)

The group outlined broad content areas to include following the study presentation.

[A book] So that we can have, for those guidelines [guiding values from the previous study] [48, 59].

(Elder Female 3)

You just gotta do an example [of the theme quotes]. Well, they've [research team] given us examples.

(Elder Female 4)

Trust and accountability within the research partnership also surfaced about the book's content and representation, with one member questioning whether their voices would be fully represented. Reassurance from the research team member and other members highlighted the shared expectation that the book would be co‐created and accurately represent participant's perspectives.

What are you going to put in? You're going to put your things in and leave some of the blackfella [an Aboriginal informal term to refer to Aboriginal people] things out.

(Elder Male 2)

No, because this comes back to you before it goes to anything. We wouldn't not include you. (Co‐facilitator 2)

They [research team] are not going to print it [the book] until we look at it.

(Elder Female 4)

These accounts reflect the importance of shared understanding of participants' ownership and control over how their knowledge is represented, reinforcing the need for trust, accountability and ongoing involvement in co‐created research processes.

The group discussed various format options, including the book size ranging from A4 to A5 and orientations including landscape or portrait. Some members preferred the A5 size for its portability, as it would fit in their bags and be easy to share with others. Some members favoured the larger A4 size. The following provides the recommendation for the book cover.

Hardcover, yeah, with an Aboriginal design on it.

(Elder Female 7)

In terms of visuals, ‘artwork’ (Elder Female 4) was mentioned as a culturally grounded mode of communication, addressing linguistic diversity and enhancing understanding across the different Aboriginal language groups.

It'll cover everything (artwork). If you do a piece of artwork, it will capture it. Whereas if you're using, as I said, the dialects differ. My dialect from Yamatji Country differs from the Noongar Country.

(Elder Female 2)

The inclusion of artwork throughout the book was noted to provide visual relief and balance within the text heavy findings. Incorporating artwork, therefore, would regulate the book length.

Well we don't expect to put a lot of stuff in it. Cause you're doing artwork and all that so.

(Elder Female 7)

As the Healthy Lifestyle Program is based within the EMHS catchment in Perth, the group centred the colours and content around Noongar Country including the ‘Noongar six seasons’ (Elder Female 1) from WA's South‐West region [60]. This reflects the importance of connecting the book in local cultural knowledge and seasonal ways of knowing. Each season represents seasonal changes, changes with local plants and animals [60] and is associated with a colour (red, orange, green, dark blue, pink and yellow) [61].

The group acknowledged that everyone has different needs, emphasising that knowledge sharing cannot be approached as a one‐size‐fits‐all process. Additional formats were recommended to broaden the book's reach and accessibility, making it available to others beyond their own records and the program to expand the knowledge sharing. The group reflected not only their preferred formats but also potential readers and the need to have multimodal versions.

But some of us don't do that [online]. I'm one of them. Computers and [QR Codes].

(Elder Female 7)

I think a lot of our kids nowadays are that way inclined…they'd sit and listen to that rather than picking up or looking at [the book] as well as listening to the DVD. But it's incorporating it because some might struggle with the booklet, but they can relate to it with the DVD.

(Elder Female 4)

More of this on your phone or something [online].

(Elder Female 5)

Many agreed that DVDs and CDs were not currently as accessible, and an online book version was more applicable. This reflects a broader shift in how engagement with research outputs is shaped by everyday technological practices, where accessibility is tied not only to availability but to relevance within participants' lived contexts. This highlights that decisions about format were not merely practical but also reflected broader considerations about usability, relational sharing and cultural relevance, including alignment with how knowledge is accessed, shared and valued in everyday life.

Unique and alternative approaches were discussed for the group to disseminate the book to other audiences and promote their involvement and the program within their communities.

…when it comes to NAIDOC Week [held in the first week in July across Australia to recognise the history, culture and achievements of Aboriginal and Torres Strait Islander Peoples], and we have that day to have a little tent or whatever‐ store, so people can come in and see this [book] and see it's out there in the community…not everyone out there in the community knows that we're sitting here doing this.

(Elder Female 4)

Future research projects recommended involving children from the program to do artwork, providing members with the opportunity to tell their own stories and including a foreword or biography for each member detailing their involvement throughout the program.

I think it'd [artwork] be great coming from the kids.

(Elder Female 4)

I'd love to see us all do little stories, but yeah that could be another book.

(Elder Female 1)

Leaving a legacy extends knowledge sharing beyond the research context, to preserving Aboriginal voices across communities and generations. Preferences for format and accessibility emphasise the importance of ensuring knowledge remains usable, culturally relevant and inclusive.

#### 3.2.2 Generational Transmission

Generational transmission refers to the process of passing values, cultural knowledge, responsibility and community leadership and traditions from one generation to the next. Many participants reflected on their own childhoods, recalling how they were shaped, guided and inspired by Elders before them.

Our Elders that have gone past, they had a lot of knowledge and a lot of wisdom to, to bring to us when we were children, you know.

(Elder Female 3)

Many now found themselves in the seats of previous generations, ‘trying to make things better’ (Elder Female 1) for their community through being part of various committees and advisory groups.

My mother and father sat on committees talking about this, and now I'm here.

(Elder Female 1)

The group expressed hope that this process would continue, with many reflecting on their own experiences now being grandparents and passing on their knowledge to younger generations. Knowledge sharing was framed as extending beyond immediate research outcomes, portraying research as part of a broader intergenerational responsibility to sustain cultural knowledge and support cultural continuity. The book served as a meaningful vehicle for sharing their experiences and contributions to research, ensuring their legacy informs and inspires future generations in research and CCI activities.

I want my grannies [grandchildren] and great‐grannies [great grandchildren] to be able to pick that book up and listen to our voices and know that we cared enough to come to meetings to try and make things better.

(Elder Female 1)

The book will tell you about my story, where I go, which meetings, and whatever. You know, so that that's like a legacy left behind, for my grandchildren and great‐grandchildren behind me, you know. And for my children as well, it'll be a wake‐up call for them.

(Elder Female 3)

Here, generational transmission is not only about knowledge, but about making visible the care, effort and commitment of being involved in research, ensuring that these are recognised, valued and carried into the future.

The group's involvement and guidance within the program and associated activities are conveyed through the metaphor of a river, symbolising the flow of knowledge, culture and well‐being across generations [59].

Like a river, from the Elders to the youth and in‐between.

(Elder Female 3)

A few of the members reflected on the composition of cultural advisory groups, noting that they often prioritise Elders and older people. For instance, the Healthy Lifestyle Program Cultural Advisory Group includes all Elders aged 60 or older. To ensure the continuity of Aboriginal knowledge, it was recommended that future generations be actively included in advisory groups to work alongside the Elders and follow in the group's ‘footsteps’ (Elder Female 3), and to continue presenting community voices in research.

It's always old people, old people getting picked. Same old, same old.

(Elder Female 7)

I hope that you're gonna bring the next generation down from us doing this.

(Elder Female 4)

These insights highlight a potential disruption in the flow of generational knowledge, suggesting that while transmission is highly valued, current advisory group practices may constrain its continuity.

### 3.3 Tips for Researchers and Continued Partnership

The group provided practical advice for researchers to build trust and collaborate effectively with Aboriginal participants and advisory groups. The tips summarise the research findings and primarily focus on cultural awareness, clear communication and the active involvement of younger generations. Table 4 summarises the tips for researchers with example quotes from the current study.

**Table 4:** Tips for researchers working with Aboriginal participants with supporting quotes.

Tips	Example quotes
Cultural awareness is paramount	‘Where we come from as a people’ (Elder Female 1)
Be accurate in reporting	‘What you're saying [participant] is then taken out of context and put in a different way’ (Elder Female 2)
Artwork can enhance understanding	‘I reckon they'd [researchers] be safer with artwork than language’ (Elder Female 2)
Give the process time	‘It's five minutes to take in a lot. You know, you can't do it [rush and cover a lot], especially when you're an Elder’ (Elder Female 5)
Keep in touch and be transparent	‘And just keeping us engaged, what's happening and that, you know?’ (Elder Female 5)
Include younger generations	‘We want the young blood in’ (Elder Female 3)

See Table 5 for details of ongoing post‐workshop involvement, including review of book drafts and artwork to ensure cultural appropriateness.

**Table 5:** Continued partnership and book development.

Activity	Description
Engagement with K.S., an Indigenous Artist and Graphic Designer (July 2025)	Collaborated with K.S. to design the book and develop the artwork inspired by the study quote: ‘Like a river, from the Elders to the youth and in‐between’ (Elder Female 3).
Exploration of online access (August 2025)	Identified Curtin University's repository as a platform for online dissemination.
Cultural Advisory Group meeting (August 2025)	Reviewed the book outline and format in relation to funding. Confirmed hard copies for the 29 Aboriginal advisors alongside an online version via Curtin University's repository. Agreed on collective authorship based on ethical guidance. Recap of the June 2025 workshop and the book draft presented; no amendments were required. Printed copies of the draft book were provided for further review with no comments or amendments suggested following T.H.'s follow‐up. Artwork elements were discussed with the group and K.S. Agreed on the Noongar six seasons as colours and the representation of the Derbal Yerrigan (Swan River) to be used in relation to the river quote.
Cultural Advisory Group meeting (October 2025)	K.S. presented the draft book artwork and the Healthy Lifestyle Program logo. Elders provided feedback, recommending the river be depicted in brown to reflect the Swan River retaining a blue outline to represent a river; the artwork was amended during the meeting with group approval. Discussed using Aboriginal wording for the program's name on the logo; however, as noted in the June 2025 workshop discussion regarding dialect variation, it was agreed to retain the title of *The Healthy Lifestyle Program*. Many members reported enjoying their involvement in the artwork and logo development. Confirmed the book format as A4 portrait, with a laminated cover and matte pages through a voting process. One member volunteered to review the Acknowledgement of Country for the book. S.S. presented the findings from this study, which the group advised captured their input. Two members volunteered to review the manuscript draft on behalf of the group.
Acknowledgement of Country review (October 2025)	Draft prepared by T.H. drawing on her experience delivering training sessions at HCCWA on Acknowledgement of Country. The draft was reviewed and approved by the group member, with a minor edit to the opening sentence.
Final book review (November 2025)	The book was reviewed by two members using a printed PDF copy from Y.C.A. Edits were made to clarify that 28 Aboriginal advisors were Elders and to acknowledge that the Cultural Advisory Group were also Elders.
Printing and dissemination (December 2025)	Books printed and made available to the 29 Aboriginal advisors.
Manuscript review (December 2025)	Two group members attended the reading of the draft manuscript of this article with S.S. and T.H.
Consumer Advisory Group meeting (December 2025)	The study findings and book development were presented to the Healthy Lifestyle Program Consumer Advisory Group with lived experience. The group echoed the study findings on the need to ensure that the book is disseminated widely and suggested posters to promote the research at general practitioners', child and parent centres and various Aboriginal health and community centres and community services. Authorship on the book was raised, and the group were advised that it was agreed to use collective terms based on ethical guidance.
Cultural Advisory Group meeting (December 2025)	Country and language groups were discussed in relation to the findings and agreed to include this information in the paper.
Cultural Advisory Group meeting (February 2026)	Verified Country and language group details with those members who participated in the June 2025 workshop. The online version was made available through Curtin University's repository [59].

## 4 Discussion

This study aimed to explore Aboriginal Elders' experiences of receiving study findings, provide guidance for researchers and develop a book that reports the findings of cultural and place‐based considerations of the Healthy Lifestyle Program for Aboriginal participants.

The current study highlighted the importance of researchers acknowledging cultural context and lived realities when working with Aboriginal participants. Having this understanding is the foundation of ethical, accurate and impactful research [62]. The Campinha‐Bacote Model of Cultural Competence has been widely used within Australia [63] including initatives undertaken in consultation with Aboriginal and Torres Strait Islander communities [64] and outlines cultural competence as a continuous and ongoing process [65]. Recognising the group members' individual Country was noted in the current study, with some of the Elders also adding their Country on the demographic forms next to the term ‘Australian Aboriginal’ which has been reported in this paper as ‘Aboriginal Australian’ [56], given Elder preference. There are over 350 different Aboriginal Nations within Australia [66], each with its own law, place, custom, language and family, making Country a fundamental aspect of identity and belief systems [26]. Including Country acknowledges place‐based identities and avoids homogenising Aboriginal and Torres Strait Islander Peoples, and recognises the diversity of communities that exist. We recommend that the language groups and Countries of individuals be collected and reported in future research, given the variance in terminology. It was also noteworthy that the group highlighted that the artwork elements for the book reflect Noongar Country where the Healthy Lifestyle Program is based, as demonstrated by including the Noongar six seasons [61] and the Derbal Yerrigan (Swan River). This highlights the importance of partnership and learning from participants and consumers for accurate, meaningful and respectful reporting of findings whenever research teams include non‐Aboriginal members.

This study exemplifies meaningful CCI and shows how CCI methods have been operationalised within a PAR approach. This was demonstrated through a focus on the principles of CCI in health and medical research. This included CCI integrated throughout the research process, mutual respect, working in partnership, equitable inclusion of diverse consumers and communities, transparency, accountability and integrity in the conduct of research, and safety of consumers and community [67, 68, 69]. CCI encourages moving beyond generic dissemination (e.g., journal articles) and instead to co‐created feedback strategies that reflect participants' preferences, cultural norms and communication needs. Embedding regular feedback loops with participants and the community (see Table 5) ensured that research findings are not only shared but also understood and supported by those who contributed to them, consistent with previous research [14, 70, 71]. The partnership and knowledge sharing among all parties (Aboriginal advisors, Cultural Advisory Group, researchers and the artist) yielded a positive outcome in the development of a culturally appropriate book that reports on cultural and place‐based considerations of the Healthy Lifestyle Program for Aboriginal participants, which can be accessed online [59]. We have made the hard‐copy version of the book available to the 29 participants.

The study findings align with a broader international body of research involving First Nations, which emphasises that knowledge sharing is not a discrete endpoint but an ongoing, relational process embedded throughout the research cycle [72]. A study from Canada highlights that Indigenous knowledge mobilisation frameworks emphasise the importance of reciprocal exchange and community leadership in determining how findings are shared, including decisions about what is communicated, through which formats and to whom [72]. PAR is widely identified as critical for ensuring cultural relevance and reciprocity [73]. These parallels suggest that the current study findings of knowledge sharing are broadly applicable across First Nations contexts. However, as shown in the current study, the specific details and modes of knowledge sharing remain place‐based and culturally grounded, highlighting the importance of locally driven approaches when translating these findings into practice [48].

In line with reflexive thematic analysis, themes were developed through iterative, reflexive engagement with participants' accounts [51, 55]. The researchers' professional, cultural and disciplinary backgrounds informed the coding process, theme generation and interpretation. Reflexivity was further enhanced through ongoing engagement with the Cultural Advisory Group, including discussion of workshop notes, sharing of interpretations and participants' involvement in reviewing the paper, which provided insight into the cultural resonance and meaning of the findings, ensuring participants' perspectives remained central throughout the analytic process.

Ultimately, the book served a dual purpose: it reported study findings while also preserving knowledge and ensuring its reach, accessibility and potential transmission across generations. The artwork and title of the book were developed around the quote ‘Like a river, from the Elders to the youth and in‐between’ conveying a story of knowledge sharing and connection, with the full artwork story accessible visually and audibly [59]. Aboriginal art is a traditional medium of storytelling and knowledge sharing, preserving knowledge, staying connected to ancestors and culture and an approach that prioritises Aboriginal voices [74, 75]. The group frequently indicated the hope that the next generation would follow in their footsteps as they had followed previous Elders. Recommendations were provided to onboard the next generation into research and advisory groups. This would include a broad range of ages for Elders to guide the process for the younger generations to ensuring that community voices and values continue to guide research. Evidence shows that Elders play an essential role in transmitting personal and cultural knowledge to younger generations, which ensures knowledge continuation [76]. Similarly, intergenerational knowledge sharing reflects a key feature of Indigenous knowledge systems globally, where knowledge is collectively held and transmitted across generations [77].

### 4.1 Strengths, Limitations and Future Research

Strengths of this study include partnership with an Aboriginal workshop facilitator and graphic designer. T.H. brought lived experience, cultural knowledge and community connections that ensured the workshop was conducted in an approach that was culturally safe, respectful and relevant. K.S., an Indigenous artist, safeguarded cultural authenticity and respect while creating visual representations rooted in tradition. Similarly, program oversight was provided from the Aboriginal Governance Group including T.H., M.R. and G.P. There is a growing awareness in the CCI literature of the urgent need to strengthen representation by including Culturally and Linguistically Diverse and Aboriginal involvement group members and younger individuals within CCI practice to guarantee that the outcomes of health and medical research are widely applicable for the benefit of all [8]. Our study supports recent CCI findings evaluating CCI Programs, indicating meaningful CCI requires renewed action to diversify participation and ensure that involvement reflects the breadth of community voices and experiences [8]. The current research and program have the continued involvement of the Cultural Advisory Group that has made this research possible. This research has included both Aboriginal participation and community involvement within a PAR approach.

A study limitation was that consent processes for both the previous and current studies required data to remain non‐identifiable. However, some of the Cultural Advisory Group wanted to be named as authors on the book. Ethical advice cautioned that naming some participants and not all could create cultural and relational concerns. In discussions with the group, it was agreed that acknowledging all contributors collectively was the most appropriate approach in this circumstance. We recommend recognising CCI representatives as authors when they meet authorship criteria and have been involved in co‐creation, co‐design and as consumer investigators, as evidenced in the literature [8, 13, 78, 79, 80], and include this intention to identify participants in ethics applications at inception. Another limitation was around the selection of the Cultural Advisory Group. The book includes the voices of the previous 29 participants; however, re‐contacting all for further engagement was not possible as funding only allowed for regular engagement with 10–12 Elders in the ongoing Cultural Advisory Group. The intention of being inclusive at the previous workshop resulted in disappointment for those not within the EMHS catchment in Perth (the area the Healthy Lifestyle Program was conducted) and were therefore not included in the Cultural Advisory Group. This approach would not be something the research team would repeat; rather, we recommend limiting initial involvement to the number able to be funded to continue with the work.

The group recommended that future research include involving children from the program in artwork, providing members with the opportunity to share their own stories (which became beyond the scope of this study), and including a foreword or biography for each member detailing their involvement throughout the program. Funding opportunities are being pursued to advance these recommendations, ensuring consent remains identifiable so the group can be recognised as authors on subsequent outputs.

## 5 Conclusion

Prioritising Aboriginal and community voices in communicating findings is essential to building and maintaining genuine partnerships with community when undertaking research. This study aimed to close the feedback loop for Aboriginal research participants. It produced a culturally appropriate book reporting findings on cultural and place‐based considerations of the Healthy Lifestyle Program.

## Notes on Language Use

The authors recognise that the term ‘obesity’ is often stigmatising. We have used this term only where necessary, including for reporting prevalence data, reproducing terminology from cited sources and orienting the biomedical audience. In other instances, we have prioritised person‐centred strengths‐based terminology.

The authors use the term ‘Aboriginal and Torres Strait Islander’ when referring specifically to the First Peoples of Australia, consistent with community guidance and national usage. We note that alternative terms, including ‘First Nations’ and ‘Indigenous’ appear in the literature and are retained when referring to cited sources. The term ‘Aboriginal’ and ‘Indigenous’ are used when referring to individuals whose terminology preferences are known. We acknowledge the diversity of Nations, languages, groups and cultural identities within Australia and internationally, and acknowledge that preferences vary between individuals, communities and across the literature.

## Author Contributions

**Stephanie Smith:** conceptualization, methodology, investigation, data curation, formal analysis, validation, visualization, project administration, supervision, funding acquisition, writing – original draft, writing – review & editing. **Tania Harris:** conceptualization, methodology, investigation, formal analysis, validation, project administration, funding acquisition, writing – review and editing. **Kelli Savietto:** visualization, validation, writing – review and editing. **Melanie Robinson:** validation, writing – review and editing. **Glenn Pearson:** validation, writing – review and editing. **Stephen Paull:** conceptualization, investigation, formal analysis, validation, project administration, funding acquisition, writing – review and editing. **Yvonne C. Anderson:** conceptualization, methodology, investigation, formal analysis, validation, project administration, supervision, funding acquisition, writing – review and editing.

## Ethics Statement

Ethical approval for the study was obtained from the Western Australian Aboriginal Health Ethics Committee (HREC1292), Child and Adolescent Health Service Human Research Ethics Committee (RGS0000006244) and reciprocal approval from Curtin University Human Research Committee (HREC2024‐0066). Research Governance approval was obtained from the Child and Adolescent Health Service Research Governance (RGS0000006244) and The Kids Research Institute Australia (P354).

## Consent

Written informed consent for the publication of anonymised participant information was obtained through a PICF. For participants who required assistance with reading, the PICF was read aloud and written consent was provided in the presence of an independent witness.

## Conflicts of Interest

K.S. was contracted to create the artwork and design the book. K.S. provided feedback on the manuscript but did not determine the results reported. The remaining authors declare that the research was conducted in the absence of any commercial or financial relationships that could be construed as a potential conflict of interest.

## Data Availability

The datasets generated and analysed during the current study are available from the corresponding author on reasonable request. The data are not publicly available due to containing information that could compromise research participant privacy/consent.
